# Modular Bayesian Networks with Low-Power Wearable Sensors for Recognizing Eating Activities

**DOI:** 10.3390/s17122877

**Published:** 2017-12-11

**Authors:** Kee-Hoon Kim, Sung-Bae Cho

**Affiliations:** Department of Computer Science, Yonsei University, 50 Yonsei-ro, Seodaemun-gu, Seoul 03722, Korea; aruwad.open@gmail.com

**Keywords:** human activity recognition, context-awareness, Bayesian network, mobile application, wearable computing

## Abstract

Recently, recognizing a user’s daily activity using a smartphone and wearable sensors has become a popular issue. However, in contrast with the ideal definition of an experiment, there could be numerous complex activities in real life with respect to its various background and contexts: time, space, age, culture, and so on. Recognizing these complex activities with limited low-power sensors, considering the power and memory constraints of the wearable environment and the user’s obtrusiveness at once is not an easy problem, although it is very crucial for the activity recognizer to be practically useful. In this paper, we recognize activity of eating, which is one of the most typical examples of a complex activity, using only daily low-power mobile and wearable sensors. To organize the related contexts systemically, we have constructed the context model based on activity theory and the “Five W’s”, and propose a Bayesian network with 88 nodes to predict uncertain contexts probabilistically. The structure of the proposed Bayesian network is designed by a modular and tree-structured approach to reduce the time complexity and increase the scalability. To evaluate the proposed method, we collected the data with 10 different activities from 25 volunteers of various ages, occupations, and jobs, and have obtained 79.71% accuracy, which outperforms other conventional classifiers by 7.54–14.4%. Analyses of the results showed that our probabilistic approach could also give approximate results even when one of contexts or sensor values has a very heterogeneous pattern or is missing.

## 1. Introduction

Recently, with the rapid development of wearable sensor environments, a human activity recognition (HAR) with consistently collected daily data and various learning classifiers has become a popular issue: a vision-based recognition using a camera [1], recognition of five daily activities with acceleration data from a mobile phone and vital signs [2], and recognition with acceleration data from a chest-wearable device [3], and so on. However, despite mature studies and analyses on simple actions, like walking, standing, or sitting, complex activities that are composed of many low-level contexts and show various sensor patterns with respect to the background contexts have not been deeply studied yet [4].

In this paper, we propose a method which recognizes the eating activities in real life. Providing automatically information related with eating activities, such as the time and duration of eating activities, is crucial for healthcare management systems for people, in general, automatic monitoring for patients, such as diabetics, whose eating activities should be carefully managed, or the elderly who live alone, and so on. Although there are already plentiful studies recognizing simple eating and other daily activities, their approach did not catch the very large variety of activities in real life and are, therefore, difficult to extend to real situations. Eating activities could be a very complicated activity to recognize using sensors, especially with limited low-power sensors, as it could have different sensor patterns with respect to different backgrounds and spatial/temporal contexts. In this paper, we propose a probabilistic method, especially the Bayesian network, which is based on the idea that those complexities might be handled better with a probabilistic approach.

The paper is organized as follows: In Section 2, we provide some analyses to show the complexity of eating activities based on the real-life logging, and specify requirements to deal with those issues. In Section 3, we explore HAR-related works using low-level sensor data, and related theories analyzing components of human activity. In Section 4, we explain how to construct Bayesian networks in further detail, and verify their realistic usefulness in a variety of angles in Section 5. Finally, Section 6 concludes the paper and discusses future works.

## 2. Background

Before further discussions, we have collected the sensor data of 10 daily activities, including eating activities, from 25 subjects (detailed specifications are provided in Section 5) equipped with the wrist-wearable device and a smartphone with sensors (see Section 4.1), and have analyzed to ascertain the complexity of eating activities and show the requirements for the eating activity recognizer to be useful in the real world.

**Figure 1:**
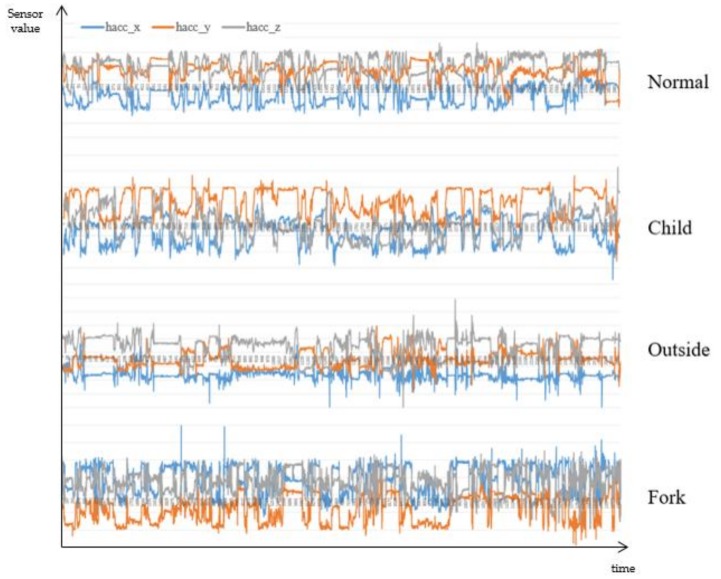
A time-series variation of acceleration sensor data in various activities.

Table 1 shows the correlation scores of each attribute with respect to the class (darker color indicates higher value). Since we had collected the various eating activities, such as eating chicken with a fork, or a sandwich with a hand, eating activities of a baby, and so on, each attribute itself showed very low correlation scores. Despite the popular adoption and relatively high performance of accelerometers, the scores of ‘h_acc’s (‘h’ for a hand, ‘acc’ for an accelerometer) are considerably low, even lower than those of the environmental attributes (‘lux’ for illuminance, ‘temp’ for temperature, ‘hum’ for humidity), except the ‘h_acc_y’ which measures the back-and-forth motion of the hand when eating. The scores of ‘acc’s are considerably high compared to other attributes, but they are also fairly low and largely caused by the constraints that the collection was not done with the user’s phone and they usually did not use the phone. Considering many people operate their smartphone while eating, it is rational to expect that those scores would be lower, like ‘h_acc’s. Table 2 shows the correlation matrix of the attributes (darker color indicates higher value), which also shows very low value, except ‘h_acc_x’ and ‘h_acc_y’, and ‘acc’s. Figure 1 shows a more specific example of a three-axis accelerometer value of the hand of four different eating activities. Even with a glimpse of observation, there are considerably different patterns: ‘h_acc_y’ of the child is comparably low as the position of the food is higher for them; the variance of all values is low when eating outside, as the user grabbed a sandwich and did not move his hand frequently; ‘h_acc_x’ is much higher than other cases when eating chicken using a fork, as the user tore on the left and right sides, and so on. In addition to the value of the sensor located on the wrist, the value of the smartphone sensor could be more unpredictable and variable as the smartphone could be anywhere while eating: in the pocket, on the table, in the hand, and so on. These could imply that the recognizer may require (i) manual modeling of activity instead of using the sensor value itself, or automatically extracted features with a learning classifier; (ii) a probabilistic reasoning that infers various kinds of contexts occurring probabilistically. In addition to the precise recognition itself; (iii) the constraint of the power and memory consumption of sensors; and (iv) the obtrusiveness to the user should be considered for the practical usage [5], as a recognizer should collect and recognize continuously without charging and too high a battery consumption could restrict the usage of devices for the original purpose.

**Figure 2:**
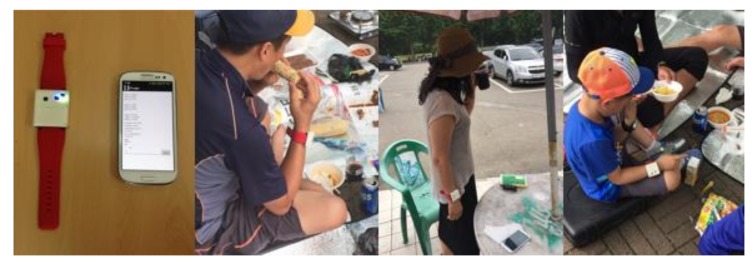
Smartphone and wrist-wearable device for data collection.

To fulfill those requirements, the proposed method (i) uses only five types of low-power sensors attached to the smartphone and the wrist-wearable device (Figure 2); (ii) is built on the context model of an eating activity which could represent the composition of complex eating activities, based on theoretical background and domain knowledge; and (iii) uses the Bayesian network (BN) for probabilistic reasoning, with a tree-structured and modular design approach to increase the scalability and reduce the cost for inference and management. Our contributions are as follows: (i) obtain and describe the complexity of real activities and the limitations of typical learning algorithms using real complex data; (ii) recognize the activity using only low-power and easily-accessible sensors; (iii) propose the formal descriptive model based on the theoretical background and show its usefulness; and (iv) provide the various experiments and analyses using a large amount of data from 25 different volunteers with 10 activities and various features.

## 3. Related Works

Approaches for human activity recognition can be classified as two categories in terms of the location of sensors: external sensors and internal sensors [5]. Using external sensors, such as surveillance cameras for intrusion detection, a set of thermometers, hygrometers, or motion detectors for a smart home, is a primary approach. However, the internal sensor approach is more suitable for eating activity recognition because (i) the external sensor approach cannot track the user as sensors are generally fixed at a specific location; (ii) a user-centered sensor environment is better than a location-centered sensor environment for personalized context-aware services; and (iii) personal sensor data could be abused for intruding privacy. For these reasons, we have chosen the internal sensor approach using a mobile and wearable device that can be widely used in daily life.

Table 3 shows recent studies of the internal sensor approach for human activity recognition using various sensors and methods. Three-axis accelerometers are most widely used for the activities deeply related with a user’s motion. However, accelerometers may not enough for the source of information when a recognizer attempts to recognize a complex activity. Bao et al. tried to recognize 20 daily activities using accelerometers attached to five locations [6]. In his experiment, accuracies of complex activities, such as stretching (41.42%), riding an elevator (43.58%), or riding an escalator (70.58%), were far lower than other simple activities, and showed larger deviations between people, or even in one person. This implies that complex activities with a great variety of different patterns may need more sensors, such as hygrometers or illuminometers, for environmental information. Cheng et al. recognized daily activities including food/water swallowing, using electrodes attached to the neck, chest, leg, and wrist [7]. Although it seems fairly reasonable using electrodes attached to the neck or chest for eating activity recognition, and they recognized various complex activities with better than 70% accuracy, their sensor environment might be uncomfortable in daily life. Obtrusiveness of the user should be concerned for the daily activity recognizer to be practical [8]. If the construction cost of the sensor environment is very high, or a user feels very uncomfortable wearing those devices, the recognizer is difficult to be used, generally. Thus, the composition and location of sensors must be acceptable for daily life. In addition, the energy consumption for sensor data collection should also be reasonable: if a smartphone will be run out of power after recognizing for just a few hours, not many people will want to use it. For this reason, it is difficult to use non low-power sensors, like the Global Positioning System (GPS) or gyroscopes.

There are also many issues for feature extraction and classification. A large number of studies used statistical indices directly calculated from the sensor data value, such as the mean, standard deviation, energy, entropy, and so on. For complex activities, like eating or drinking, manual observation for patterns has also been conducted [7]. As shown in Figure 1, and studies in Table 3, sensor values could have a large deviation between people with various ages, genders, cultures, or even in one person. We attempted to find and construct the general context model for activity recognition based on the “Five Ws” (who, what, when, where, and why) and activity theory. The Five Ws are a publicly well-known and self-explanatory method to analyze and explain a situation for humans, so it can give a more understandable result [11]. Marchiori attempted to classify a very large amount of data on the World Wide Web based on Five Ws, and Jang used the Five Ws to define a dynamic status of a resident in a smart home [11,12]. Although the Five Ws give us a systematic and widely-agreed method of describing a situation, it is too abstract to apply directly to low-level sensor data. For example, eating a lunch at a restaurant cannot be directly recognized by acceleration or temperature. It should be embodied in a measurable level like ‘correspondence of the space illumination’. Activity theory gives more specific evidence on how an activity should be composed. Nardi compared an activity theory with situated action models and a distributed cognition approach to systemically understand a structure of human activity and situation [13]. According to activity theory, a human activity consists of a subject, which includes human(s) in that activity, an object as a target object of the subject, which induces a subject to a special aim, an action that subject must perform in order to achieve the intended activity, and an unconsciously and repetitively occurring operation while doing an activity [14]. While action theory is primarily to examine the individual’s own behavior as an analysis unit, situated action theory focuses on the relevance of actors and environmental factors at the moment of occurrence of the activity [15,16]. According to this theory, defining a human activity systemically should sufficiently consider environmental factors which can fluctuate dynamically [13]. In our proposed model, subject properties represent emergent properties of an eating person, which can be subclassified as an action and an operation. To deal with environmental factors, we use spatial and temporal properties independently.

For the classifiers for human activity recognition, learning approaches, such as decision trees, hidden Markov models, naïve Bayes, and nearest neighbor, are dominant. A large number of studies show a high accuracy for many daily activities (Table 1). However, as an activity becomes complex, or the number of subjects increases, many deterministic classifiers may not give good accuracy: Tapia et al. recognized various exercising activities and obtained over 90% accuracy for one subject, but 50–60% for many subjects. Vinh et al. used a probabilistic approach, a semi-Markov conditional random field, and showed good accuracy for complex activities, including dinner, lunch, and so on [10]. In this paper, we propose the Bayesian network that learns its conditional probability table for the probabilistic approach.

## 4. Proposed Method

**Figure 3:**
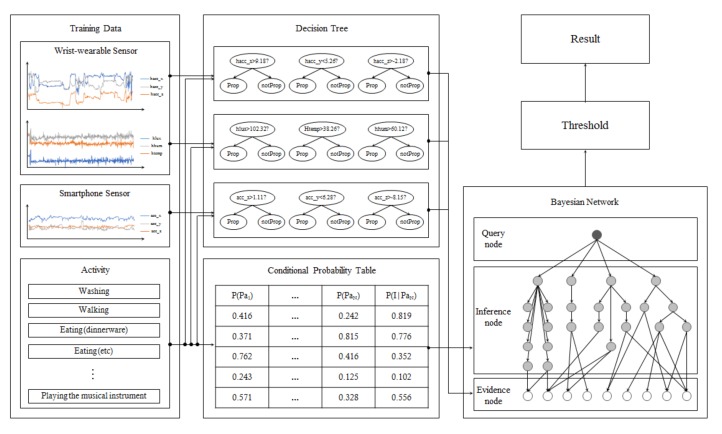
An overview of the proposed method.

Figure 3 shows the overall system architecture of the proposed method. It has a modular BN that infers the target activity node from a child node, which infers the low-level context, and simple decision trees that infer evidence nodes of the modular BN (see Section 4.2 and Section 4.3). When the training process starts and the raw sensor data from nine channels and its class information are entered, the system learns and constructs its decision tree and conditional probability table, as described in the Section 4.3. For the recognition, the trained decision trees obtain raw sensor data continuously and make an inference of the probability of their evidence node, and the modular BN infers gradually from the evidence nodes to the query node, the eating activity. If the probability of the query node is larger than the predefined threshold, the recognition result becomes ‘eating’.

### 4.1. Sensors

As mentioned in Section 1, we only used low-power sensors attached to the smartphone and a wrist-wearable device to consider constraints of power consumption and obtrusiveness of the user. The distribution rate of the wrist-wearable device is much higher than other forms of wearable devices and is in a natural position to collect daily life data consistently. Moreover, as we use our hands to eat something, the wrist is an appropriate position to collect food intake-related movement and the position of hands, and parametric temperature or humidity. We combined the four kinds of sensors for the wrist-wearable device (Figure 2), which are composed of MPU-9250 motion sensor of InvenSense (Seoul, republic of Korea), BME280 environment sensor of Bosch (Seoul, republic of Korea), and APDS-9900 illumination sensor of Avago Technologies (Seoul, republic of Korea). Table 4 shows the type of sensors with their power consumption and collecting frequency. The device can collect data continuously for about 6 h without charging.

### 4.2. Context Model of Activity

An eating activity is a complex activity which consists of many low-level contexts, such as the spatial and temporal background, movement of the wrist, and temperature. Table 5 shows the web ontology language (OWL) representation of the proposed context model based on the activity theory and the “Five W’s”, for systemic analysis on an eating activity. Four subclasses represent the components of the Five W’s, except ‘Why”, as this context is considered difficult to measure with the limited sensor environment. A subject property consists of goal-directed processes (actions) and the unconsciously appearing status of the body (body temperature, posture, and so on; operations). Nine properties describe the low-level context of the eating activity. Each intermediate node is linked to leaf nodes, namely, sensors, which are considered as related. Although the movement of the user is the main feature to recognize activities, used for most intermediate nodes, environmental features could also contribute, especially when the movement patterns are diverse. The proposed context model has three other subclasses (object, spatial, and temporal properties) to consider those environmental factors. A temporal property uses the system time for judging one property, whether the current time is appropriate for eating. A spatial property has four properties, such as whether the user is indoors or outdoors, changes of space, and whether the intensity of illumination of the space is appropriate for eating.

### 4.3. The Proposed Bayesian Network

A formal definition of the BN and its nodes are as follows.

**Definition 1.**  *A BN is a directed acyclic graph (DAG) with a set of nodes N, a set of edges $E=(N_{i},N_{j})$ , and a conditional probability table (CPT) which represents a causal relationship between connected nodes. Each node represents a specific event on the sample space Ω, and each edge and the value of the CPT represent a conditional relationship between a child node and parent nodes, P(C=c|P=p). Given the BN and evidence e, the posterior probability P(N|e) can be calculated by chain rule, where Pa(N) is the set of parent nodes of N [17]:*

(1) $$P\left(N|e\right)=\prod P\left(N|Pa\left(N\right)\right)\times e=\prod P(N|Pa\left(N\right))\prod_{e_{i}\in e}e_{i},$$

**Definition 2.**  A set of nodes N consists of the set of query nodes Q, which represents the event user wants to know from the BN a set of evidence nodes V, which observes the sensor data and classifies the properness, and a set of inference nodes I, which infers the probability of related contexts based on a CPT.

**Figure 4:**
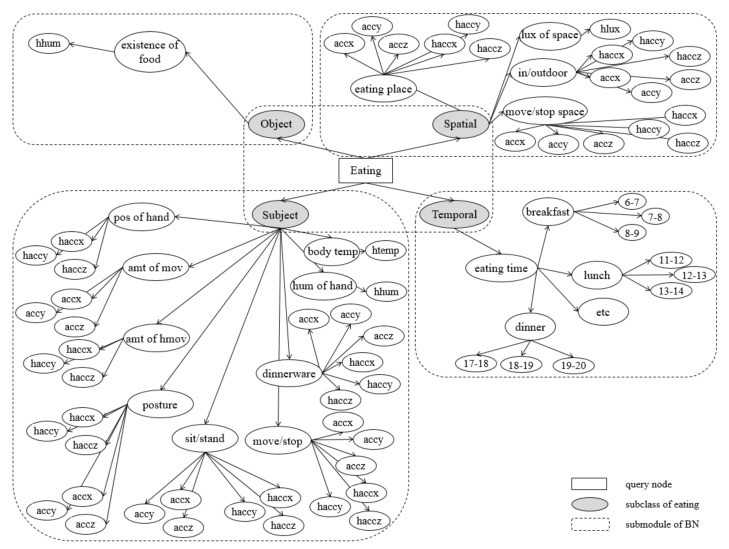
The proposed Bayesian network.

Figure 4 shows the proposed BN. The proposed BN consists of *V*, *I*, and *Q*, where |V|=64, |I|=23, and |Q|=1. Full names of sensors are described in Table 4. Nodes in *V* are set by nine types of low-level sensor data, the query node in Q represents the recognition result, eating or not, and each intermediate node in *I* represents the sublevel context of the target activity. By using intermediate nodes, the proposed model is more resistant to overfitting than typical learning models which mainly depend on automatically calculated statistics, such as the mean, deviation, or Fourier coefficients. For example, even if the model is trained only with the eating data using a fork, it could approximately recognize the eating activity using chopsticks if the user eats while sitting and shows the similar pattern of the movement of the hand, and so on. Moreover, in addition to the complex composition of the eating activity itself, there could be many unexpected or omitted sensor values: user may eat while lying down or eat at midnight, or take off the wrist-wearable device or smartphone, where the accelerometer value is omitted. A BN could deal with these issues as it provides the probabilistic approach for recognizing each context, so it can give an approximate answer even if some data are uncertain or missing, compared to other deterministic classifiers which give a wrong answer or cannot give any answer at all.

For a structure of the proposed BN, we construct the modular BN with a tree-structured design.

**Definition 3.**  Modular Bayesian network [18]. A Modular BN (MBN) consists of a set of submodular BNs M and the conditional probability between submodules R. Given BN submodules $\theta_{i}=\left(V_{i},E_{i}\right)$ and $\theta_{j}=\left(V_{j},E_{j}\right)$, the link $R_{i,j}=\{<\theta_{i},\theta_{j}>|i\neq j,V_{i}\cap^{\text{​}}V_{j}=\emptyset \}$ is created. Two submodules are connected and communicate only by shared nodes.

The proposed MBN has one main module containing a query node and four submodules where each leaf node in a main module (object/spatial/subject/temporal) becomes the root node of each submodule. All submodules are designed by a tree-structured approach, where each module has only one root node, which is also a shared node, and all child nodes have exactly one parent node. By following these design approaches, the proposed model is more explainable as the probability of each shared node could easily be calculated and explain the probability of each context to an individual. Moreover, these design approaches substantially reduce the complexity of the BN to $O(k^{3}n^{k}+wn^{2}+(wr^{w}r^{w})n)$; by limiting *k* to 2 and minimizing the *w*, where *n* is the number of nodes, *k* is the maximum number of parents, *r* is the maximum number of values for each node, and *w* is the maximum clique.
**Algorithm 1.** Learning algorithm for the CPT.for∀D,// D is the input data
increment numOfData by 1;
C :=class of D;for i=1 to n(I) do $\mathrm{if}\;C\;\mathrm{includes}\;I_{i}\;\mathrm{then}$     $\mathrm{increment}\;\mathrm{num}\left(I_{i}\right)\;\mathrm{by}\;1;$
     $\mathrm{if}\;\exists \;q\;\in \;Q\;s.t.\;q\;\in \;C\;\text{then increment num}(I_{i}\cap Q);$for i = 1 to n(I) do
 $P\left(I_{i}\right):=\frac{num\left(I_{i}\right)}{numOfData};$
       $\mathrm{CPT}\left(I_{i}\right):=P\left(I_{i}|Q\right)=\frac{P\left(I_{i},Q\right)}{P\left(Q\right)}=\frac{num\left(I_{i}\cap^{\text{​}}Q\right)}{num\left(Q\right)};$

To calculate the value of the CPT, the proposed BN learns the data using simple learning algorithm. In the training process, the training data enters into *E* and *I*. For evidence nodes in *E*, there is a simple binary decision tree for each evidence node and it learns a criterion for classification. For inference nodes in *I*, BN counts the number of occurrences that $C\subset I_{i}\;\mathrm{for}\;\forall I_{i}\in I$ and update the element of the CPT, as shown in Algorithm 1. For example, if $C_{k}=\left\{sitting\right\}\cap^{\text{​}}\left\{dinnerware\right\}\cap^{\text{​}}\left\{eating\right\}$, $C_{k}\subset I_{1}=\left\{sitting\right\}\;\mathrm{and}\;C_{k}\subset Q_{1}=\left\{eating\right\},\;\mathrm{so}\;num\left(I_{1}\right)\;\mathrm{and}\;num\left(I_{1}\cap^{\text{​}}Q_{1}\right)$ increment, and so on. For this algorithm, the proposed BN needs O((M+N)×ND) time complexity for learning, where ND is the amount of data, and when either the number of nodes or data is fixed, the time complexity becomes linear.

## 5. Experimental Results

### 5.1. Data Specification

For the experiment, we collected 948 min of data from 25 different volunteers for 10 activities. Subjects were asked to wear a wrist-wearable device and have a smartphone, performed activities that they wanted to perform, and tagged the activity they were doing on the smartphone when the new activity started. They were also asked not to perform more than one activity simultaneously to collect accurate sensor data for each class. If they performed another activity that were not supposed to be collected, such as moving to another place or getting a phone call, collection was temporarily stopped. To collect as much real-life data as possible, we did not request them to come to a certain place; instead, we went to where they lived while performing their daily activities and collected the data. When a self-tagging was difficult, like for a baby or the elderly who are not familiar with a smartphone, we observed and tagged their activities simultaneously. Each subject performed, at most, four different activities and each activity was prolonged for, at most, 20 min to prevent a small number of subjects from dominating most of the data. A specific distribution of each item is shown in Table 6, and indices of activities and jobs are shown in Table 7. We attempted to balance the gender of the subjects, and chose the list of activities by referencing Activities of Daily Livings (ADLs) which is known as a proper method describing the functional status of a human, performing an important role in a healthcare service [19]. ‘Etc’ in the job includes a four-year old baby. An eating activity consists of 47.27% (448 min out of 948 min), so the data is well-balanced in terms of the eating activity.

Table 8 shows a brief comparison of the collected data with other popular open data for HAR: Opportunity dataset [20] and Skoda dataset [21]. Note that as our approach is supposed to recognize various real eating activities with people with various contexts, we focused on collecting the data from a sufficiently large number of subjects, so the length of collected data for each subject is relatively small, which is supposed to capture short intervals of daily life, mainly including eating activities. Additionally, note that we tried to use very limited sensors and devices, which are supposed to only include low-power sensors that are easy to use in daily life.

### 5.2. Accuravy Test

**Figure 5:**
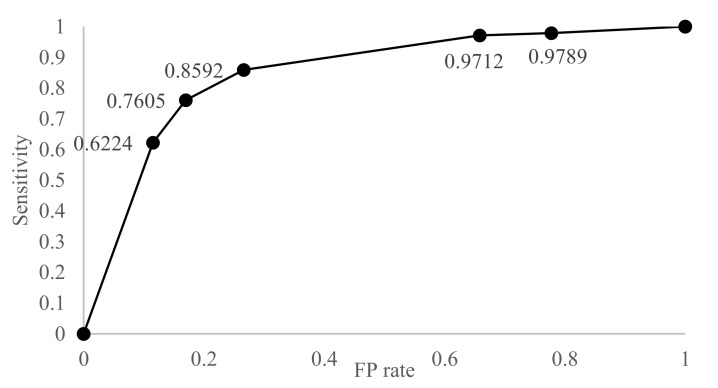
ROC curve for the proposed BN.

**Figure 6:**
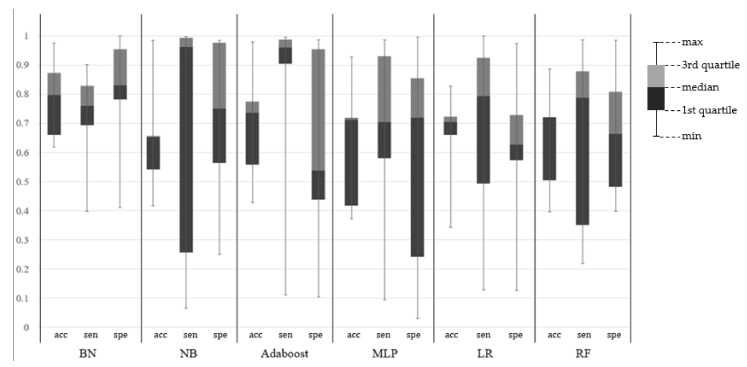
Ten-fold cross-validation for other typical classifiers (accuracy, sensitivity, specificity).

Table 9 and Table 10 show the result of the 10-fold cross-validation of the proposed BN. The proposed BN produced 76.86% accuracy with the threshold value of 0.6. The specificity of the proposed BN (83%) was higher than the sensitivity (76.05%), which means that the proposed BN classifies better in the non-eating activity than the eating activity. Figure 5 shows the ROC (receiver operating characteristic) curve as the threshold for the eating probability decreases. The cost for decreasing the threshold was the smallest at the point ‘threshold = 0.6’, and where the threshold is lower than 0.2, the BN classified all activities as an eating activity. As shown in Figure 5, the AUC (area under curve) is fairly large, which supports the usefulness of the BN. Figure 6 shows the accuracy, sensitivity, and specificity of the various typical learning classifiers. We used the Weka 3.8.0 tool (of the university of the Waikato, Hamilton, New Zealand) to analyze the results. Five classifiers have a large deviation between tests, as they tend to be overfitted to the train data; when the test data is composed mostly of similar data with the train data, their performance is very high, but in the other case, they are very low. The proposed BN, LR, and RF showed smaller deviations. The accuracy of the proposed BN was 7.54–14.4% higher than other classifiers. In the case of naïve Bayes and Adaboost, sensitivities are very high (96.15% and 95.91%, respectively), but specificities are also very low (37.68% and 53.77%, respectively), which means that the two classifiers classified most cases as an eating activity. For the multilayer perceptron (MLP), it showed good results among five other classifiers, but the time to build the model and classify was much higher than other methods. For the one-sample t-test, suppose the population has a normal distribution, and let the null hypothesis $H_{o}=\prime accuracy<0.8\prime$. With $\bar{X}=0.7854,s=0.386,t=-0.0378>-2.262,\;\mathrm{and}\;H_{o}$ is rejected. When $H_{o}\prime {=}^{\prime }accuracy>0.9^{\prime },t=-0.2969<-2.262$, so $H_{o}\prime$ is rejected and the proposed model is expected to have an accuracy of 0.8–0.9 for the population.

### 5.3. Error Case Analysis

**Figure 7:**
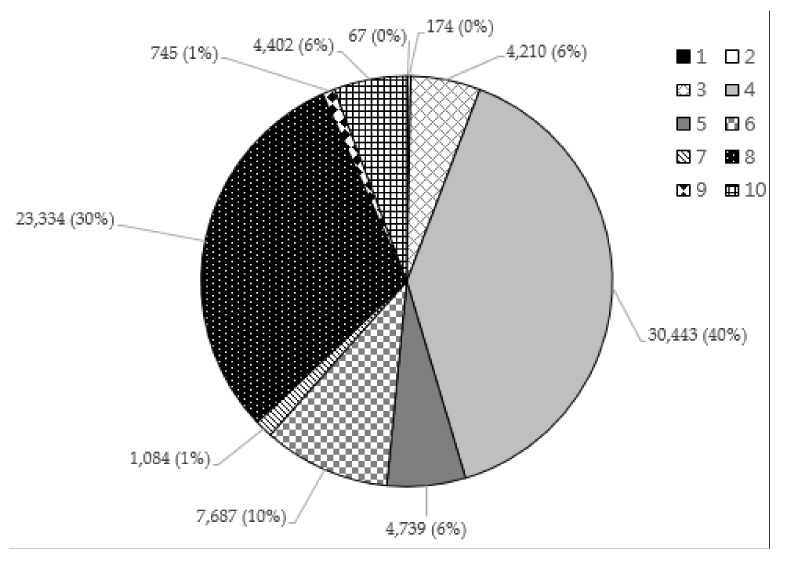
Proportion of the error case.

**Figure 8:**
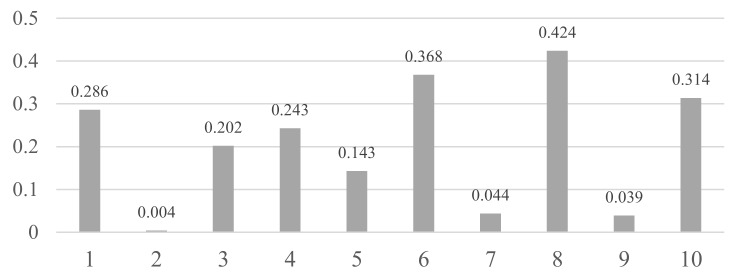
Error rate of each activity.

Figure 7 shows the proportion of each activity to the whole error case, and Figure 8 shows the error rate of each activity. The index of each activity is shown in Table 7. Eating with dinnerware shows the highest proportion (40%), followed by sedentary work (30%) and conversation (10%). However, due to the proportion of eating with dinnerware being far greater than that of sedentary work, the error rate is much larger with respect to sedentary work (0.424). As sedentary work and conversation generally show similar patterns in the amount of movement of the hand, and usually happens indoors, the same as with the eating activity, the two activities show a higher error rate than any other activities. However, in the case of walking, as it is typically a dynamic activity easily distinguished from the eating activity, it showed a very low error rate (0.004%; 174 lines out of 39,822 lines). For driving and subway activities, differences of movement and spatial properties make those activities’ error rates low.

**Figure 9:**
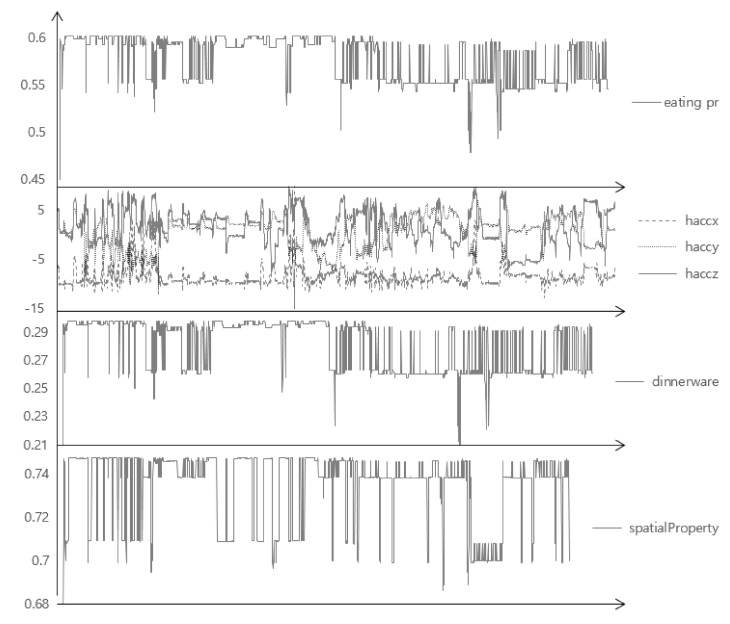
Eating activity of a left-handed person.

Figure 9 shows the specific case, which is the eating activity of a left-handed person, who wore the wrist-wearable device on the right wrist and mainly used the left hand to eat, but also used the right hand for moving food, using a smartphone, gesturing in conversation, and so on. Compared to the right-handed person (Figure 1), the accelerometer shows a different pattern, such as a much lower and steady value for the *x*-axis and a higher and irregular pattern of the *y* and *z*-axis, as they used their right hand for various purposes in addition to eating. As a result, the probability of using dinnerware shows very low and high deviance. However, as the person ate in a normal environment like other subjects, the spatial property compensating the final recognition and overall eating probability shows acceptable results. This means that the proposed BN could approximately recognize the complex eating activity when one of the contexts or sensor values has a very different pattern or is even omitted. Note that the proposed method might approximately recognize these cases without incorporating information of which hand the person uses and applying different algorithms. This is important since, in the real world, the person might use different hands for various situations; one might prefer to use the left hand to drink coffee, while using the right hand to eat chicken.

## 6. Conclusions

In this paper, we proposed the eating activity recognition method based on a Bayesian network, using low-power sensors attached to a smartphone and a wrist-wearable device. Contributions of this paper are as follows: (i) obtain and describe the complexity of real activity and limitations of typical learning algorithms using real complex data; (ii) recognize it using only low-power and easily-accessible sensors with low time complexity; (iii) propose the probabilistic model based on the theoretical background; and (iv) provide the various experiments and analysis using large data from 25 different volunteers for 10 activities and various features, showing the usefulness of the proposed method. The proposed method showed an accuracy of 79.71%, which is higher than other learning classifiers, with of 7.54–14.40% better accuracy. We analyzed the error case and the results show that the proposed method could approximately give the answer even when some of contexts or sensor values are very different. Future works include the collection of much larger and representative data, the construction and evaluation of the proposed method for various complex and daily activities, and the evaluation of the proposed method with open data.

## Figures and Tables

**Table 1:** Correlation scores of each attribute.

Name ^1^	Value	h_acc_x ^2^	h_acc_y	h_acc_z	h_lux	h_temp	h_hum	acc_x	acc_y	acc_z
Correlation	Pearson correlation coefficient	0.1068	0.2887	0.0819	0.0217	0.0101	0.1379	0.2351	0.2837	0.3997
InfoGain	H(C)−H(C|A)	0.0883	0.1866	0.0725	0.0685	0.1202	0.1556	0.4786	0.4604	0.336
GainRatio	$\frac{H\left(C\right)-H(C|A)}{H\left(A\right)}$	0.0142	0.0304	0.0137	0.0133	0.0157	0.02	0.076	0.0678	0.0737
SymUncert	$\frac{2\left(H\left(C\right)-H\left(C|A\right)\right)}{H\left(C\right)}+H\left(A\right)$	0.0245	0.0523	0.023	0.0222	0.0278	0.0354	0.1311	0.1181	0.1208

^1^ Correlation coefficient, information gain, information gain ratio, symmetric uncertainty; ^2^ h = hand, acc = accelerometer, lux = illuminometer, temp = temperature, hum = humidity.

**Table 2:** Correlation matrix of attributes.

	h_acc_x	h_acc_y	h_acc_z	h_lux	h_temp	h_hum	acc_x	acc_y	acc_z
h_acc_x	1	0.32	0.07	0.04	0.08	0.03	0.09	0.08	0.15
h_acc_y		1	0.1	0.07	0.16	0.07	0.13	0.19	0.21
h_acc_z			1	0.04	0.05	0.04	0.04	0.12	0.14
h_lux				1	0.06	0.07	0.17	0.04	0.05
h_temp					1	0.09	0.21	0.23	0.22
h_hum						1	0.01	0.06	0.02
acc_x							1	0.49	0.61
acc_y								1	0.77
acc_z									1

**Table 3:** Sensors, activities, and methods of daily activity recognition works.

Author	Sensors	Activities	Feature Extraction	Classifier
Jatoba et al. [5]	Accelerometer (wrist, elbow, etc.)	Walking, jogging, climbing upstairs, etc.	Step count, mean value of local maxima, angle value, etc.	K-nearest neighbor, naïve Bayes, binary decision tree, etc.
Bao et al. [6]	Accelerometer (wrist, ankle, tight, elbow, hip)	20 daily activities (eating, walking, etc.)	Mean, energy, entropy, etc.	Decision tree, naïve Bayes, nearest neighbor, decision table
Cheng et al. [7]	Electrodes (neck, chest, leg, wrist)	Looking to various sides, bread/water swallowing, etc. (while sitting/walking)	Manual observation, time-domain features	Linear discriminant analysis
Tapia et al. [9]	Accelerometer (right-wrist, tight, ankle), heart rate monitor	Various exercise (walking, running, ascending/descending stairs, cycling, rowing, etc.)	Mean distance, entropy, correlation coefficient, FFT peaks and energy	Decision tree, naïve Bayes
Lee et al. [10]	Accelerometer (wrist, hip)	20 daily activities (dinner, lunch, office work, etc.)	Mean, standard deviation, mean crossing rate	Semi-Markov conditional random field

**Table 4:** Sensors attached to wrist-wearable devices for recognition.

Sensor	Abbreviation	Units	Power Consumption	Collecting Frequency
Accelerometer	h_acc	m/s^2^	450 µA	20 Hz
Illuminometer	h_lux	lux	250 µA	1 Hz
Thermometer	h_temp	°C	1.0 µA	1 Hz
Hygrometer	h_hum	g/m^3^	0.8 µA	1 Hz

**Table 5:** OWL representation of the context model for eating activity recognition.

Class: Eating activity	subClassOf: Subject property	subClassOf: Activity	subClassOf: Wrist	ObjectProperty: Position of hand
				ObjectProperty: Dinnerware
				ObjectProperty: Movement of hand
			subClassOf: Body	ObjectProperty: Posture
				ObjectProperty: Move/stop
				ObjectProperty: Movement of body
		subClassOf: Operation	ObjectProperty: Body temperature	
			ObjectProperty: Posture	
			ObjectProperty: Humidity of hand	
	subClassOf: Object property	ObjectProperty: Existance of food		
	subClassOf: Spatial property	ObjectProperty: Eating place		
		ObjectProperty: Indoor/outdoor		
		ObjectProperty: Move/stop		
		ObjectProperty: Illuminance of space		
	subClassOf: Temporal property	ObjectProperty: Eating time		

**Table 6:** Data specification.

Activity	Count	Job	Count	Gender	Count
1	1 (4%)	1	3 (12%)	M	12 (48%)
2	2 (8%)	2	2 (8%)	F	13 (52%)
3	1 (4%)	3	1 (4%)	Age	Count
4	11 (44%)	4	6 (24%)	0~10	2 (8%)
5	6 (24%)	5	1 (4%)	20~30	9 (36%)
6	3 (12%)	6	8 (32%)	30~40	2 (8%)
7	2 (8%)	7	3 (12%)	40~50	3 (12%)
8	5 (20%)	8	1 (4%)	50~60	8 (32%)
9	1 (4%)			60~	1 (4%)
10	1 (4%)				

**Table 7:** Index of activities and jobs.

Index	Activity	Job
1	Washing	Undergraduate
2	Walking	Graduate
3	Housework	Student
4	Eating (dinnerware)	Houseworker
5	Eating (etc.)	No job
6	Conversation	Office worker
7	Driving	Businessman
8	Sedentary work	etc.
9	Subway	
10	Playing the piano	

**Table 8:** Comparison of our dataset with another open dataset for HAR.

	Number of Subjects	Number of Instances	Length	Activities	Sensors
Our dataset	25	379,013	16 h	10 daily activities	Three-axis accelerometers (2), hygrometer, illuminometer, thermometer
Opportunity	4	96,667	6 h	17 simple activities	Inertial measurement unit (7), three-axis accelerometers (12)
Skoda	1	179,853	3 h	10 gestures	Three-axis accelerometers (20)

**Table 9:** Confusion matrix of the proposed BN.

	Positive	Negative
True	TP = 136,354	FN = 42,937
False	FP = 33,949	TN = 165,773

**Table 10:** Statistical indices of the results.

Index	Value
Accuracy	$\frac{TP+TN}{TP+TN+FP+FN}\;=79.71\%ficity\;includes\;4-year\;oldsincludes\;4\;-year\;olds\;baby)recognition\;using\;accelerometer.\;nodes\;and\;E,\;and\;a\;set\;of\;query\;nodes$
Precision	$\frac{TP}{TP+FP}=80.07\%$
Sensitivity	$\frac{TP}{TP+FN}=76.05\%$
Specificity	$\frac{TN}{FP+TN}=83\%$
